# P02.192. The mediating role of bodily dissociation and emotion regulation on PTSD symptoms among women in substance use disorder treatment

**DOI:** 10.1186/1472-6882-12-S1-P248

**Published:** 2012-06-12

**Authors:** C Price, J Herting

**Affiliations:** 1 University of Washington, Seattle, USA

## Purpose

Dissociation from the body involves a sense of separation from bodily self and typically involves a lack of attention to sensory awareness. Dissociation from the body is common among women with a history of sexual trauma and interferes with emotion regulation. Interoceptive awareness (inner body awareness and processing), has recently been posited as important to homeostasis and regulation to facilitate relapse prevention among individuals in substance use disorder treatment. The purpose of this study was to explore the mediating role of bodily dissociation and emotion regulation on PTSD symptoms among women in substance use disorder treatment.

## Methods

The sample (N=46) were women in substance use disorder treatment participating in a NIH-funded study to examine a body-oriented therapy intervention that teaches interoceptive skills. The majority of the sample (~70%) had been exposed to the body-oriented therapy intervention. We used a SEM modeling approach with maximum likelihood estimation to test the effect of bodily dissociation and emotion regulation on PTSD symptoms across the 9 month study period involving assessments at baseline, 3, 6 and 9 month follow-up. The Scale of Body Connection, Difficulties in Emotion Regulation, and Modified PTSD Symptom Scale were used.

## Results

The results showed significant indirect effects of bodily dissociation and emotion regulation on PTSD symptoms. Specifically, the mediation model indicates the following temporal relationship between variables: bodily dissociation → emotion regulation difficulties → PTSD symptoms where bodily dissociation has no direct effect on later PTSD symptoms but operates through its effect on emotion regulation difficulties.

## Conclusion

The results suggest the role of bodily dissociation reduction in emotional regulation among women seeking treatment for a substance use disorder that have a history of sexual trauma. These findings support current theoretical models specific to interoception for relapse prevention, and point to the need for interventions that teach interoceptive skills.

